# Is educational expansion beneficial for health? A counterfactual mediation analysis of its impact on trends of daily activity limitations among different age groups in Germany

**DOI:** 10.1186/s13690-026-02051-6

**Published:** 2026-08-25

**Authors:** Batoul Safieddine, Johannes Beller, Stefanie Sperlich

**Affiliations:** Department of Medical Sociology, Hannover Medical School, Hannover, Germany

**Keywords:** Social inequalities, Activity limitations, Educational expansion, Germany, School education, Trends

## Abstract

**Background:**

Education has been a well-established indicator of health. Against the background of educational expansion, little is known on how the temporal rise in educational attainment is affecting health trends. This study mainly examines the mediation effect of educational expansion on the trends of limitations in daily activity as measured by the global activity limitation indicator (GALI).

**Methods:**

The study utilized data from the German Health Update survey (GEDA) using five waves between 2008 and 2024. Predicted probabilities and trends of (severe) limitation in daily activities were estimated using logistic regression analyses. Educational inequalities in the rates of limitation in daily activities in the five waves as well as their development over time were examined using the slope and relative indices of inequalities (SII, RII). Causal mediation analyses were applied to examine how educational expansion influenced the temporal development of (severe) limitations. All analyses were stratified by gender and three age groups.

**Results:**

The results reveal significant disparities in severe limitations in daily activities, particularly widening among the middle-aged group. The findings show divergent trends in limitation across age groups: older individuals have experienced considerable improvements, while younger individuals have experienced an increase in severe limitations over time. In both cases, educational expansion was found to have a positive mediating effect on the temporal development of (severe) limitations, reducing the exacerbation in younger individuals and contributing to the improvement in older individuals.

**Conclusion:**

Educational expansion appears to have a positive influence on health trends, which reinforces the need for continued educational reforms. More focus is needed on younger individuals to explore factors behind their declining health. Additionally, addressing widening educational inequalities, particularly among middle-aged adults, is crucial for narrowing the gap between educational groups. Social challenges and the digital divide could be important contextual factors to address this development.

**Supplementary Information:**

The online version contains supplementary material available at 10.1186/s13690-026-02051-6.

**Table:** 

Texbox 1. Contributions to the literature
• This study expands understanding of education as a dynamic social determinant of health by examining how rising educational attainment contributes to long-term trends in self-perceived limitations in daily activities.
• It identifies vulnerable population groups by revealing divergent age-specific trends, with worsening activity limitations among younger adults alongside improvements among older adults, and widening educational inequalities especially in middle-aged adults.
• It also strengthens the evidence base for equity-oriented education policies and reforms by highlighting the importance of reducing educational inequalities and promoting higher educational attainment.

## Background

Education has long been recognized as a key determinant of health and wellbeing, with numerous studies demonstrating its positive impact on both physical and mental health outcomes. Higher levels of education are consistently associated with a lower risk of chronic diseases [1, 2], improved self-rated health [3] and increased life expectancy [4–6]. One of the most established mechanisms through which education promotes health is through literacy, which enables individuals to make informed health choices, adopt healthier lifestyles, and navigate healthcare systems more effectively [7, 8]. In addition, higher educational attainment is linked to better mental health, with evidence suggesting that it helps decrease stress and access to resources, and develop skills and networks that enhance the ability to cope with health risks [9]. Other pathways through which education influences health also include a higher social class and a better control over life [10]. Additionally, the benefits of education on health extend beyond the individual level, with populations with higher educational attainment generally exhibiting better public health outcomes [6].

The association between education and health has been proposed to reflect a causal relationship. A substantial body of economic research has examined whether education has a causal effect on health outcomes, which stems from the concerns that the well-documented educational gradients in health may reflect selection rather than a true causal relationship. For example, using quasi-experimental designs based on compulsory schooling laws, several studies provide evidence that additional schooling can lead to improvements in health outcomes while considering key confounding factors. Lleras-Muney (2005) finds that these additional years of schooling significantly reduce adult mortality in the United States, providing some of the strong evidence that education itself contributes to better health [11]. Another study based on combined data from three nationally representative surveys from fourteen OECD countries used variation in the timing of educational reforms as a construct for education and showed that the association between education and health is causal, with a significant improvement in different subjective health measures associated with one additional year of schooling [12]. Additionally, a study from the UK examined the causal effect of education on chronic health conditions, and showed that educational reforms that raised minimum school leaving age and policy changes that improved educational attainment led to a significant reduction in the probability of having diabetes [13]. Thus, the level of education achieved appears to be a key resource for life opportunities in society and plays a significant role in determining overall health.

In the last few decades, a positive development of educational attainment has been observed in various populations, including Germany. The significant growth in educational involvement with individuals spending more time in school and achieving higher qualifications is labeled as *educational expansion* [14]. In Germany, educational expansion has been taking place since the education reforms during the 1960s and 1970s. This implies that a greater number of young people are pursuing advanced education and earning higher degrees. In Germany, with the presence of three different types of schooling that lead to different academic and career paths, the shift is clearly illustrated by comparing the types of schools attended then and now. In the 1950s, the majority of students (79%) went to the “Hauptschule”, which is the school type designed for practical, hands-on learning, but whose certificate does not permit higher university education or an equivalent [15]. In 2018, this proportion went down to around 30%, which was compensated with a notable increase in the proportion of people attaining a university entrance qualification [16]. This expansion has had substantial social and societal impacts, influencing areas like the education system itself, the types of jobs available, the employment landscape, and the evolvement of social standards and beliefs [14, 17]. Subsequently, and beyond these broad societal effects, the rise in higher educational attainment also has personal consequences, affecting individuals' health and their opportunities in life.

Given the crucial role education plays in health, it is highly relevant to examine the impact of educational expansion on the way health and wellbeing are developing over time. Research examining the temporal development of objective as well as subjective health outcomes is abundant. The temporal development of objective health outcomes has been examined in studies that illustrate differential trends depending on the outcome, population or subgroup examined [18–20]. While considering objective health outcomes is essential for prevention and health policy, subjective health also plays a significant role in this context. This is because it also considers psychosocial well-being accompanying illness, which can greatly differ depending on personal, societal and environmental aspects. Longitudinal studies have shown that subjective health is a valid predictor of health outcomes [21], with evidence on it having an stronger effect compared to objective health in predicting mortality [22]. Studies from countries with advanced economies considering various subjective health outcomes mostly display an improvement of health over time [23–26], while negative trends as reported by some studies cannot be ruled out completely. Nevertheless, evidence also points towards differential trends, or varying levels of improvement of subjective health, depending on the age group considered [23]. Apparently, older individuals have been benefiting more from the positive health trend than younger individuals, with some evidence on worsening of subjective health in the younger population [27, 28].

While the effect of education on health outcomes has been widely studied, the broader question of how educational expansion would affect the temporal development of health and wellbeing over time remains to be under-investigated. A study from Germany by Klein et al. analyzed data from the German socioeconomic panel between 1984 and 2004 and concluded that higher education levels over time are associated with increased life expectancy. Healthier behaviors, more flexible income opportunities, continuous career paths, and lower work-related health burdens were proposed as possible contributors to this positive association [29]. Similarly, a systematic analysis of the effects of increased educational attainment on child mortality in 175 countries concluded that 51.2% of the reduction in child mortality between 1970 and 2009 could be attributed to educational expansion [30]. Nevertheless, most existing research focuses on mortality as an outcome, and overall, only a few studies examined the effect of educational expansion on indicators of subjective health, highlighting the fact of insufficient research on this topic.

This study aims to participate in filling the gap by examining the extent to which educational expansion influences the temporal development of self-perceived limitation of daily activity due to health problems in a gender and age group stratified analysis of nationally representative samples of the German population. By doing so, the study would be contributing to understanding the effects of educational expansion on public health, identifying vulnerable population groups with respect to self-perceived activity limitation, defining mechanisms through which subjective health develops over time and proposing hypotheses for potential mediators for the effect of educational expansion on health to be examined in future research.

Specifically, this study aims to answer the following research questions:

1. How is self-perceived limitation of daily activity due to health problems developing over time in men and women of three age groups?
2. How does this development differ among different educational groups?
3. How are potential educational inequalities in activity limitations developing over time in the subgroups considered?
4. Does educational expansion have a mediating role in the temporal development of activity limitations among the subgroups considered? If yes, to what extent?

Given available evidence from Germany described above, we hypothesize that subjective health as measured by self-perceived activity limitations is improving with a significant mediating role of higher educational attainment. However, the positive development and the mediating role of education would vary in extent depending on the age group considered.

## Methods

### Data

The German Health Update study (German: Gesundheit in Deutschland Aktuell [GEDA]) is a nationwide cross-sectional survey of the resident population living in Germany. It has been regularly conducted since 2008 on behalf of the Federal Ministry of Health by the Robert Koch Institute (RKI) and is part of the health monitoring at RKI. In addition to health status, GEDA collects information on health behaviors, living conditions, and the utilization of healthcare services. GEDA is a continuous survey that enables the establishment of a flexible surveillance instrument at RKI. GEDA has been widely used in previous research to examine a broad range of health outcomes [31–34] and health-related behaviors [35–38] in the German adult population, both in cross-sectional analyses and in trend analyses over time. In this article, the data from the waves of 2009, 2012, 2019, 2021, and 2023 were used. These waves were chosen based on the availability of the variables in need and the similarity of the survey method. The survey period and case numbers, as well as descriptive statistics, are provided in Table 1. The surveys considered in this study were conducted using structured questionnaires (Computer Assisted Telephone Interview (CATI)). The study is based on a random sample of landline and mobile phone numbers in all waves included (dual-frame method). Following the EU-level framework regulation for European statistics, the target population in GEDA includes the German-speaking adult population living in private households, whose usual place of residence at the time of data collection was in Germany. This definition excludes collective households such as hospitals, nursing homes, retirement homes and guesthouses. For sample selection, a telephone sampling system, which allows for near-complete coverage of the target population, was used. The data collection was carried out by external market and social research institutes. RKI staff supervised the entire data collection process through continuous supervision and comprehensive field monitoring [39].

**Table 1:** Descriptive characteristics of the study populations in five German Health Update survey waves conducted between 2008/2009 and 2023/2024

Wave	2008/2009	2012/2013	2019/2020	2020/2021	2023/2024
Period	07.2008—05.2009	02.2012—3.2013	04.2019—03.2020	04.2020—09.2020 & 06.2021—12.2021	01.2023—02.2024
N	21,262	19,294	14,654	13,084	31,972
Women* %	51.5	51.3	50.4	51.2	51.1
Age*					
18–39 years %	33.0	31.1	26.7	31.1	31.5
40–64 years %	42.7	44.3	43.5	40.7	40.4
65 + years +	24.2	24.6	29.7	28.1	28.1
School Education* %					
Low	36.5	33.4	29.5	26.6	26.3
Middle	37.9	36.5	38.7	39.3	39.7
High	25.6	30.1	31.8	34.1	34.0

^*^weighted according to population weights of the corresponding waves

Generally, GEDA waves involved recruiting data during two calendar years (Table 1). Thus, the waves used in the study were renamed to include both years in which the survey was done to provide accuracy. The wave 2019 involved recruiting data during the period 04.2019 – 09.2020. To separate effects of the lockdown during the COVID-19 pandemic, we included participants of this wave until March 2020, where the COVID-19 lockdown had started in Germany. Participants in this wave who participated between April and September 2020 were added to the participants of the following wave. Thus, the wave 2019–2020 is free from any COVID-19 effects, and the wave 2020–2021 in this study could be defined as the COVID-19 wave, where results should be interpreted within this context.

### Education

Education was categorized based on the highest level of schooling attained. Individuals with a maximum of 9 years of schooling were classified in the "low education" group. This category also includes individuals who did not complete any school-leaving certificate. The "middle education" level encompassed individuals with 10 years of schooling with earning a corresponding school certificate. Individuals with at least 12 years of schooling, and who obtained a certificate granting university entry, were assigned to the "high education" group.

### GALI

Self-perceived activity limitation was examined through the global activity limitation indicator (GALI). GALI measures the respondents’ self-assessment of whether they are limited in their daily activities by a physical, mental or emotional health problem, including disease or impairment, and old age, given that these limitations have been ongoing for at least six months. Individuals with long-term health-related limitations typically go through an adaptation process which affects their daily life activities. GALI serves as a measure to evaluate the severity of these limitations based on standards of the corresponding population with its cultural and social expectations [40].

Other subjective health measures such as self-rated health have been considered to examine the temporal development of health inequalities [34]. The present study extends this body of work by incorporating GALI, thereby adding a complementary perspective that captures long-term, health-related activity limitations. Moreover, the validity of GALI has been assessed against measures of disability, activities of daily living and functional limitations and has been shown to strongly correlate with these across different European countries with adequate levels of validity and reliability [41, 42]. The response options are divided into three levels to distinguish the severity of activity limitations: severely limited (severe limitations), limited but not severely (moderate limitations) and not limited (no limitations). In this study, the GALI variable was dichotomized by combining the responses for severe and moderate limitations into a single category, "limited" (coded as 1), with the second category being "not limited" (coded as 0).

### Stratification

Since self-assessment of health, as well as the effect of education on health outcomes might be greatly influenced by gender and age, the analyses in this study were stratified by gender and three age categories. To examine age-specific patterns while maintaining sufficient sample size within each stratum, analyses were stratified by three broad age groups: 18–39, 40–64, and 65 + years. Extensive research on different health outcomes differentiates between younger, middle-aged, and older adults using similar age boundaries [43–45]. These categories reflect meaningful life stages in adulthood, young adulthood (early working life and family formation), middle age (peak workforce participation and increasing chronic health risks), and older adulthood (higher prevalence of functional limitations and retirement).

### Time trend

For examining the temporal development of activity limitations, school education and the temporal development of educational inequalities in limitations, time trend was examined through a categorical variable representing the five survey waves, with the first wave (2008–2009) serving as the reference category.

### Statistical analyses

First, we examined the temporal trends in the three levels of school education across the five time periods. Logistic regression analyses were applied to this investigation. The dependent variable was the level of education, with separate models for each gender and age group. The main independent variable was the categorical time trend, with wave one serving as the reference category. All models were adjusted for age within each age group and weighted according to the provided population weights for each wave to enhance representativeness of the data with respect to age, gender, region and education. Based on these regression analyses, predicted probabilities, which represent prevalence rates adjusted for the included covariates, were estimated using margins at means to report educational level rates across the different waves.

Second, the development of GALI was analyzed using the same approach. Here, GALI, categorized as 0 (not limited) and 1 (limited), served as the dependent variable, with time wave as the independent variable and wave one as the reference category. The models were adjusted for age within each age group and weighted according to the population weights. Next, to further explore the temporal development of health across different educational levels, this analysis was stratified by education.

Third, we examined educational inequalities in GALI and the temporal development of these inequalities using the Relative Index of Inequality (RII) and the Slope Index of Inequality (SII) measures [46]. The RII measures the ratio of the prevalence between individuals at the extremes of the educational spectrum, in our case the lowest and highest educational levels. SII captures the absolute health inequality between those educational levels. As a prerequisite to calculating these measures, ridit scores, which represent cumulative rank probabilities, were calculated for each educational group, age group and gender. Following standard methodology of this approach, a logarithmic link function and an identity link function were applied to compute RII and SII, respectively [46]. First SII and RII were examined for each considered subgroup within each of the examined waves. Second, to examine whether educational inequalities are widening or narrowing over time, we added a two-way interaction term between the ridit scores for education and the categorical time variable with the first wave being the reference category. The reference category was “high education in 2008–2009”. Interaction terms with RII > 1 and SII > 0 indicate widening health inequalities, disadvantaging individuals with the lowest educational attainment.

Finally, we conducted causal mediation analyses to assess the extent to which educational expansion is associated with changes in health over time. Causal mediation analyses is a counterfactual framework that allows for inferring causal conclusions of mediation mechanisms under a strict set of assumptions that consider confounding factors at different levels [47]. This method is commonly applied to assess intervention or trial effects mediated through a certain factor, comparing outcomes between treated and controlled groups [48]. In the present study, however, we apply causal mediation analysis within a counterfactual framework to decompose observed differences in activity limitations between the 2008–2009 and 2023–2024 survey waves into components attributable to changes in educational composition of the population and to other time-related factors. Educational expansion is not conceptualized as a discrete treatment, but as a population-level mediator reflecting a gradual process of educational expansion over time. Accordingly, the exposure variable represents survey wave rather than a specific exposure or intervention, and the analysis does not aim to estimate causal effects in the classical sense. Because the compared populations differ across multiple social, political, and contextual dimensions that cannot be fully captured by measured covariates, key assumptions underlying causal mediation analysis, particularly sequential ignorability and non-parametric assumptions, are not applicable and unlikely to be fully satisfied. The causal mediation framework was nonetheless chosen over classical mediation approaches because it accounts for exposure–mediator interaction by applying a counterfactual estimation of trends of activity limitations while fixing the baseline distribution of education, allowing direct and indirect effects to vary across mediator and exposure values [49]. Results are therefore interpreted as descriptive and theory-informed estimates of how activity limitation trends might have differed in the absence of educational expansion, rather than causal effects.

In our mediation analyses, we used logit models where the exposure was a binary variable of time wave, with the two categories: wave 2008–2009 “0” versus wave 2023–2024 “1”. The mediator was the binary variable of high education “1” versus low and middle education “0”, and the outcome was the binary variable of having (severe) self-perceived daily activity limitations “1” versus having no limitations “0”. Separate mediation models were applied for each age group and gender, resulting in six mediation models. In each mediation model, age within the age group was adjusted for in the embedded mediator and outcome models. Also, similar to all performed analyses in the study, we adjusted in all models for population weights of the considered waves to correct for the sampling design bias and make wave-samples more representative of the corresponding populations in terms of age, gender, region and education.

The key estimated coefficients include total, direct and indirect effects of the exposure on the outcome. The total effect (TE) represents the overall effect of the independent variable on the outcome without any simulated effects of the mediator: TE = E[Y(1,M(1))] − E[Y(0,M(0))]. The natural direct effect (NDE) reflects the effect of the independent variable on the outcome that remains after blocking the effect of the mediator, i.e. the difference in activity limitation between 2008–2009 and 2023–2024 with the counterfactual scenario that the mediator, educational distribution (high versus low and middle) remained constant over time: NDE = E[Y(1,M(0))] − [Y(0,M(0))]. The natural indirect effect (NIE), captures the portion of the effect of the independent variable on the outcome that is mediated by the mediator, as measured by NIE = E Y_i_ [1, M(1)] − Y [1, M(0)]. A graphical representation of the mediation model is provided in Additional file 1.

## Results

The number of participants in the considered waves ranged from 13,084 to 31,972, with women comprising slightly more than half in each wave. The age distribution varied considerably across the waves, indicating the need for age stratification in the analyses. Basic demographic information is found in Table 1.

### Educational expansion

**Fig. 1:**
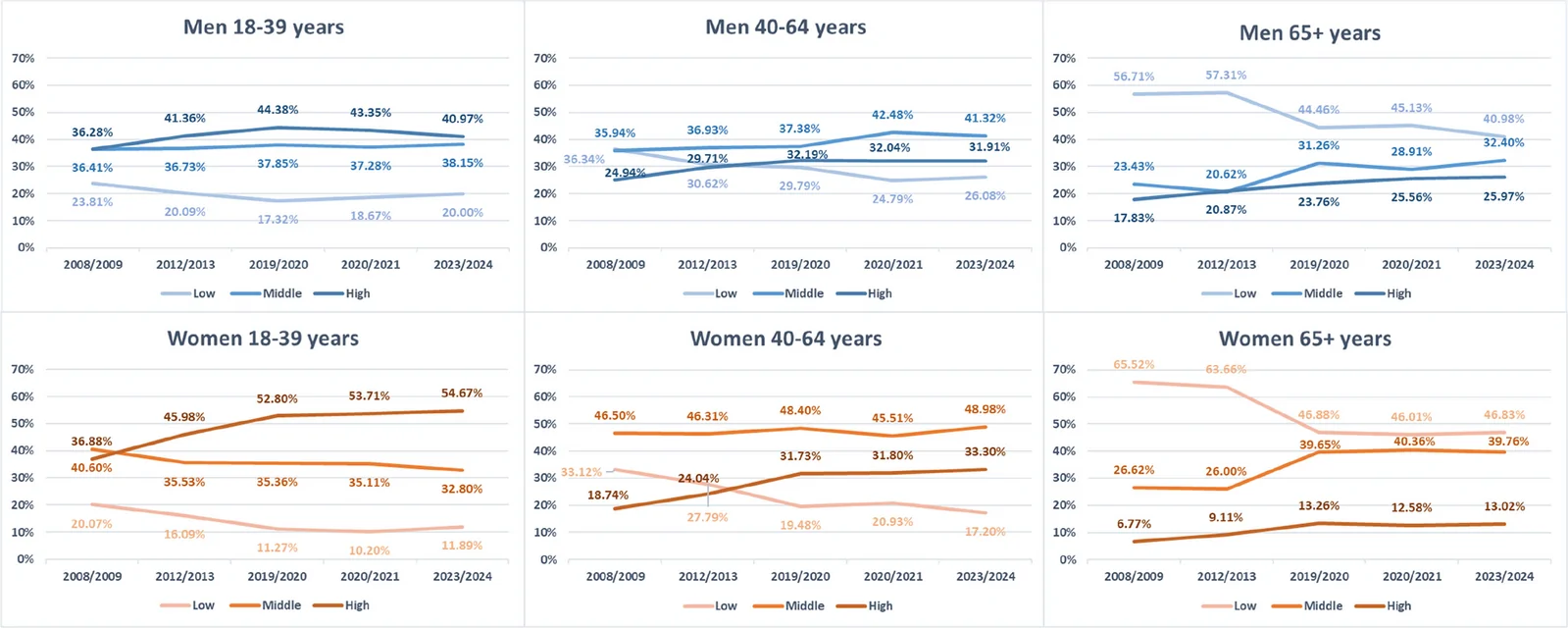
Predicted probabilities of low, middle and high education displaying trends of school educational attainment levels and educational expansion between 2008/2009 and 2023/2024 among adults in Germany, stratified by gender and three age groups. Examined by means of logistic regression analyses, adjusted for age within each age group and weighted according to population weights

Predicted probabilities for the various levels of school education across different waves confirm an overall educational expansion for both genders and all three age groups. For example, among individuals aged 18–39 years, the age-adjusted predicted probability of low education decreased by 3.81%-points for men and 8.18%-points for women between the waves 2008/2009 and 2023/2024. This decline in low education was offset by an increase in the predicted probabilities for higher education for both genders. The educational expansion became more pronounced in older age groups, as evidenced by a substantial decrease in the probabilities for low education, accompanied by a significant rise in both high and middle education levels (Fig. 1 & Additional file 2).

### Trends of GALI

**Fig. 2:**
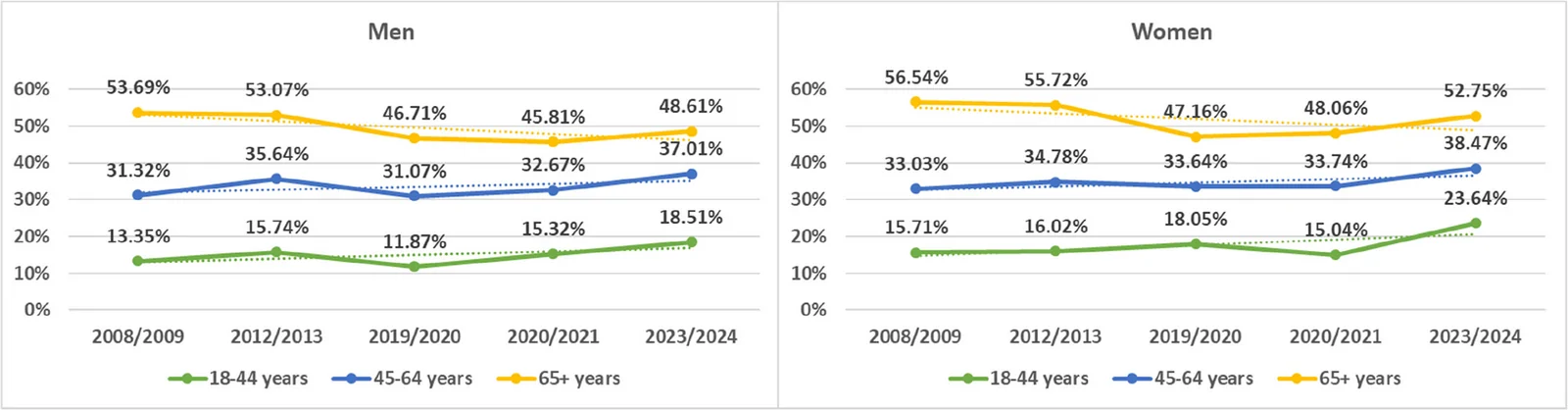
Predicted probabilities of activity limitations displaying trends over time among adults in Germany, stratified by gender and three age groups. Examined by means of logistic regression analyses, adjusted for age within each age group and weighted according to population weights

The overall trend in GALI, as shown by the temporal development of being limited versus not limited, reveals distinct patterns across age groups. For individuals aged 65 + years, the predicted probabilities of experiencing (severe) limitations decreased for both men and women. In contrast, the probabilities increased in the two younger age groups, with a sharper rise in the 18–39 years age group, especially among women (Fig. 2 & Additional file 2).

**Fig. 3:**
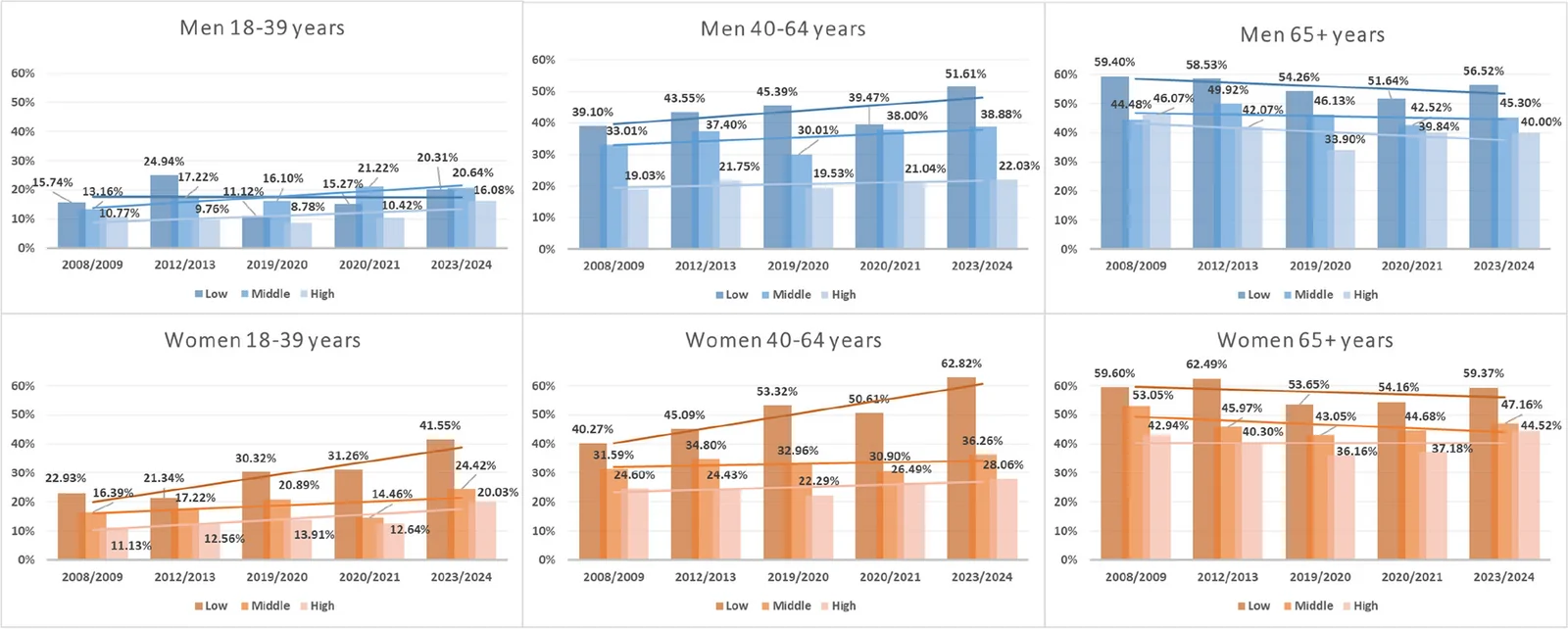
Predicted probabilities of activity limitations displaying trends over time among adults of different educational groups in Germany, stratified by gender and three age groups. Examined by means of logistic regression analyses, adjusted for age within each age group and weighted according to population weights

When stratifying the analyses by education, the above-described temporal development pattern for being limited could be largely reproduced. Predicted probabilities for experiencing (severe) limitations increased in all three education groups in men and women of the two younger age groups. In the age group 65 + years, the reduction in the predicted probabilities between 2008/2009 and 2023/2024 for being limited was observed only in the low and high education group in men and the middle education group in women. Notably, education gradients were observed in almost all subgroups and waves, with individuals with lower education having higher predicted probabilities for experiencing (severe) limitations (Fig. 3 & Additional file 2).

### Education inequalities in GALI

The slope and relative indices of inequality indicated significant educational inequalities among all waves. In the waves 2008/2009 and 2012/2013, all examined subgroups had significant inequalities, with lower education groups having significantly higher odds for experiencing (severe) limitations. In the following waves, men aged 18–39 years did not show significant relative inequalities. In the wave 2020/2021, which represents the wave strongly influenced by covid-19 and lockdown measures, only middle-aged men and women had significant relative inequalities in (severe) limitations. Men and women with low education had RII values of 2.05 and 2.18 respectively, indicating an over two times the rates of being limited in daily activities compared to their high education counter parts (Table 2).

**Table 2:** Slope Index of Inequality (SII) and Relative Index of Inequality (RII) in activity limitations by educational level among adults in Germany across five survey waves from 2008/2009 to 2023/2024, including temporal trends in both inequality measures, stratified by gender and three age groups. Estimates were adjusted for age within each age group and weighted using population weights

		Men 18–39 years			Men 40–64 years			Men 65 + years			Women 18–39 years			Women 40–64 years			Women 65 + years		
		n	Index	95% CI	n	Index	95% CI	n	Index	95% CI	n	Index	95% CI	n	Index	95% CI	n	Index	95% CI
2008/2009	SII	3113	0.06*	0.01, 0.10	4152	0.25**	0.20, 0.30	1673	0.22**	0.14, 0.31	3903	0.13**	0.09, 0.18	5659	0.14**	0.09, 0.18	2272	0.15**	0.08, 0.23
	RII		1.84*	1.19, 2.84		2.26**	1.84, 2.76		1.72**	1.36, 2.16		2.70**	1.95, 3.73		1.71**	1.42, 2.06		1.30*	1.06, 1.58
2012/2013	SII	2470	0.14**	0.09, 0.19	4490	0.29**	0.24, 0.34	2238	0.21**	0.14, 0.29	2344	0.09*	0.03, 0.15	4555	0.19**	0.14, 0.24	2923	0.21**	0.15, 0.28
	RII		3.86**	2.42, 6.25		2.19**	1.82, 2.64		1.58**	1.29, 1.95		2.07*	1.26, 3.41		2.02**	1.63, 2.51		1.69**	1.39, 2.06
2019/2020	SII	1270	0.13*	0.05, 0.20	2812	0.30**	0.23, 0.36	2745	0.28**	0.21, 0.35	982	0.17*	0.06, 0.27	3145	0.34**	0.27, 0.40	3269	0.19**	0.13, 0.26
	RII		1.84	0.99, 3.44		3.00**	2.23, 4.03		1.90**	1.52, 2.37		3.08*	1.57, 6.06		2.90**	2.20, 3.81		1.64**	1.31, 2.04
2020/2021	SII	924	0.13*	0.04, 0.23	2274	0.29**	0.22, 0.37	2425	0.28**	0.21, 0.36	758	0.12	0.00, 0.24	2677	0.27**	0.20, 0.34	2877	0.16**	0.09, 0.22
	RII		2.27	0.92, 5.61		2.05*	1.29, 3.26		1.32	0.92, 1.89		2.48	0.70, 8.85		2.18*	1.28, 3.69		1.37	0.96, 1.97
2023/2024	SII	989	0.11*	0.01, 0.21	1885	0.38**	0.30, 0.47	1968	0.22**	0.14, 0.30	784	0.18*	0.03, 0.32	2395	0.26**	0.18, 0.33	2473	0.16**	0.09, 0.23
	RII		1.42	0.74, 2.73		2.85**	2.11, 3.85		1.59*	1.22, 2.06		2.37*	1.14, 4.95		2.66**	1.92, 3.68		1.42*	1.14, 1.78
Temporal development of educational inequalities measured through interaction between time and education—Reference category: high education in 2008–2009																			
2012/2013	SII	8766	0.08*	0.02, 0.15	15,613	0.05	-0.02, 0.12	11,049	-0.01	-0.12, 0.10	8771	-0.05	-0.12, 0.03	18,431	0.05	-0.02, 0.11	13,814	0.08	-0.02, 0.18
	RII		1.59	0.96, 2.66		0.97	0.78, 1.22		0.95	0.74, 1.20		0.74	0.46, 1.18		1.08	0.88, 1.32		1.13	0.93, 1.37
2019/2020	SII		0.07	-0.02, 0.15		0.04	-0.04, 0.12		0.04	-0.07, 0.15		0.03	-0.08, 0.15		0.19**	0.12, 0.27		0.02	-0.07, 0.12
	RII		1.53	0.78, 3.00		1.36*	1.03, 1.79		1.08	0.86, 1.37		1.19	0.63, 2.23		1.81**	1.44, 2.28		1.08	0.89, 1.31
2020/2021	SII		0.08	-0.02, 0.18		0.03	-0.05, 0.12		0.05	-0.06, 0.16		-0.01	-0.13, 0.12		0.13*	0.05, 0.21		0.001	-0.10, 0.10
	RII		1.64	0.78, 3.42		1.30	0.97, 1.76		1.17	0.91, 1.49		0.93	0.45, 1.91		1.69**	1.31, 2.18		1.04	0.85, 1.27
2023/2024	SII		0.07	-0.03, 0.18		0.13*	0.04, 0.22		0.002	-0.11, 0.12		0.03	-0.12, 0.18		0.12*	0.03, 0.20		0.02	-0.08, 0.12
	RII		1.24	0.65, 2.36		1.34*	1.02, 1.76		0.96	0.75, 1.22		0.87	0.47, 1.63		1.28*	1.01, 1.63		1.00	0.82, 1.21

^*^p < 0.05

^**^p < 0.001

The temporal development of education inequalities in being limited in daily activities as examined by double sided interactions of education and time indicated that inequalities are widening over time, especially in men and women of the age group 40–64 years (Table 2). As can also be observed in Fig. 3, educational inequalities have increased over time in this age group with trend lines illustrating a considerably wider gap between educational levels in the wave 2023/2024 compared to the earlier waves. The Covid-19 period 2020–2021 appears to be associated with fluctuation in the temporal development of educational inequalities. Still, in the last compared to the first examined period, which encompass around 16 years, widening inequalities remained to be statistically significant (Men: SII = 0.13; RII = 1.34, *p* < 0.05; Women: SII = 0.12; RII = 1.28, *p* < 0.05) (Table 2).

### The mediating role of education

**Fig. 4:**
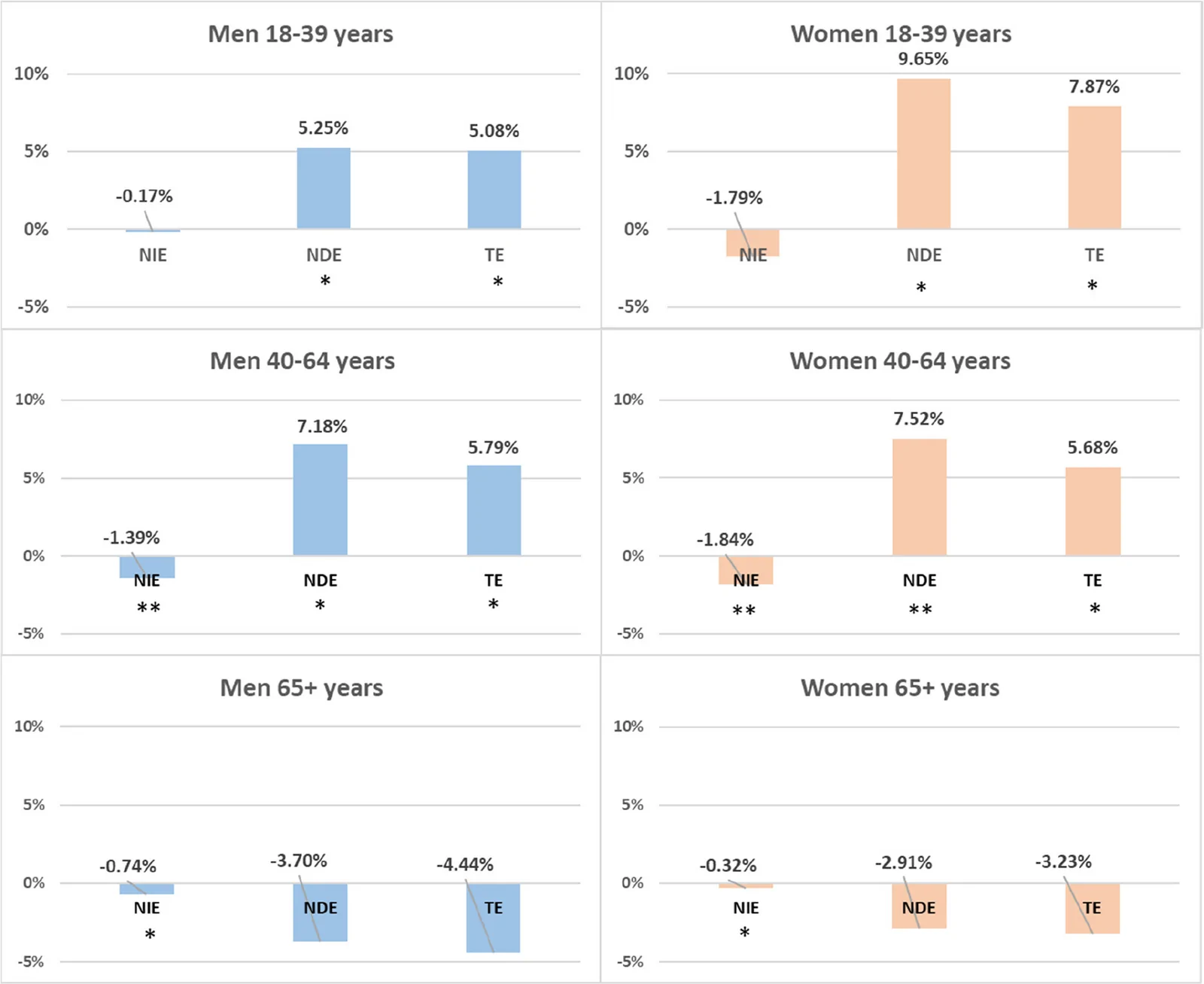
Mediation effects of educational expansion on the temporal development of activity limitations among adults in Germany using causal mediation analyses, stratified by gender and three age groups. Adjusted for age within each age group. NIE: natural indirect effect; NDE: natural direct effect; TE: total effect. *p < 0.05; **p < 0.001

Similar to the results displayed above, the causal mediation analyses revealed the differential temporal development of being limited in daily activities among the different age groups. A positive significant total effect of time wave on being limited in the two younger age groups indicates an increase in (severe) limitations in these subgroups, while a negative total effect in the age group 65 + years indicates a decline of (severe) limitations over time in this subgroup (Fig. 4). The causal mediating analyses also indicated that educational expansion in this population had a positive mediating effect on the temporal development of experiencing (severe) limitations in all three age groups and both genders. In men and women of the age group 18–39 years, the mediation analyses displayed a significant deterioration in activity limitations. In women, there was a total increase of 7.87%-points of self-perceived activity limitation. A 1.79%-points less increase is attributed to educational expansion, indicating that without educational expansion, the increase in activity limitation would have been more pronounced in this subgroup. In men, the significant increase in activity limitation was less pronounced compared to women but still statistically significant, with a 5.08%-points increase between the first and the last period. Educational expansion was associated with 0.17%-points less deterioration in activity limitations in this subgroup. Even though educational expansion showed a positive mediating effect, the NIE attributed to educational expansion is “statistically” non-significant for both men and women of this age group. In men and women aged 40–64 years, the rates of limitations in daily activities also increased over time, and educational expansion had a highly statistically significant and positive mediating effect, illustrating that the increase in rates would have been more pronounced in the absence of educational expansion. As illustrated by the NIE in the models, educational expansion prevented an additional 1.39%-points and 1.84%-points potential increase of rates of experiencing (severe) limitations in men and women, respectively (*p* < 0.001). In the age group 65 + years, the decline in the rates of experiencing (severe) limitations in daily activities was also significantly mediated by education, with 0.74%-points and 0.32%-points decrease attributed to educational expansion in men and women, respectively (*p* < 0.05). Even though educational expansion has a significant role in the temporal development of health in most subgroups, the NDE in all models indicates that the majority of the change in (severe) limitations over time is attributed to other factors.

## Discussion

The positive development of educational attainment was evident in this study with the increasing rates of individuals with higher education over time. This educational expansion has been shown to have a significant positive mediating effect on the temporal development of self-perceived activity limitation: In the absence of educational expansion, increases in activity limitations would likely have been more pronounced in younger age groups, while the decline observed among older adults would have been weaker. While studies examining the effect of educational expansion on subjective health outcomes are scarce, evidence on the positive effects educational expansion has on other health outcomes such as mortality is in line with our results [29, 30]. This study added that the positive effects also apply when considering a well-established and valid subjective health indicator that reflects limitations in daily activities due to chronic health problems, GALI. Nevertheless, although the estimated mediation effects are statistically significant, their magnitude is small in absolute terms, typically below two percentage points. In standardized terms, these effects correspond to a small fraction of a standard deviation of the outcome. As educational expansion is a gradual, long-term societal process whose social and behavioural consequences for health unfold over extended periods and across generations, modest effect sizes are not unexpected. Still, these findings should be interpreted as evidence of a clearly detectable mediating role of educational expansion in shaping trends in activity limitations, rather than as strong causal mediation effects, which would require a more robust causality examination. Moreover, although temporality between education and activity limitations is plausible, given that school education is typically completed before adulthood, our study design does not allow for causal inference. We therefore emphasize that the use of causal mediation analysis in this study does not imply a causal interpretation in the classical sense. Educational expansion is conceptualized as a gradual societal process unfolding over time, embedded in broader social, political, and contextual changes that distinguish between the 2008–2009 and 2023–2024 populations. As such, key assumptions of causal mediation analysis, including sequential ignorability [48], are not applicable and unlikely to be fully met. We used a causal mediation framework as an analytical tool for trend analysis, allowing us to examine how observed trends in activity limitations might have differed under a counterfactual scenario in which the educational composition of the population had remained unchanged. Our findings should therefore be interpreted as descriptive and theory-informed, rather than as evidence of a causal mediation effect. The potential “causal” pathway between educational expansion and improvement in activity limitations needs to be examined in further research.

Several contextual factors could play a mediating role between educational expansion and better health. Structural factors such as income or occupational position [50], psycho-social factors such as perceived social support [51] and social participation [52] as well as lifestyle factors such as nutrition and physical activity [53, 54] could all be mediating the causal pathways between educational expansion and wellbeing. Accordingly, the positive effect could be explained by educational expansion providing more individuals with greater opportunities for personal growth, skill development, and socioeconomic mobility. For example, as access to education increases, people might gain better employment opportunities, which often leads to improved financial stability, a key factor in overall wellbeing and life satisfaction. Furthermore, education enhances critical thinking, social connections, and a sense of empowerment, all of which contribute to better health related behavior and physical and mental health outcomes. Evidence suggests that higher education is associated with better life satisfaction, as it equips individuals to overcome challenges, make informed decisions, and engage in beneficial social and professional relationships [55]. Moreover, higher education has been linked to health literacy [7, 8], which is a key determinant of lifestyle choices that greatly impact health and wellbeing. Nevertheless, studies examining these potential mediators for the association between education and health within the context of educational expansion are scarce. This calls for further examination against the background of educational reforms in different societies to better understand the mechanisms that lie behind the temporal development of health outcomes.

The results of the study also highlighted the notable differential trends of the rates of experiencing (severe) limitations in daily activities in younger and older individuals, with educational expansion having a positive impact on both trends. Part of the improvement in daily activities limitations among older individuals was attributed to educational expansion, but also, the worsening of (severe) limitations in younger individuals would have been more pronounced if it had not been for educational expansion. These differential trends are not unique to this study, as similar patterns have been observed in other research [23, 27, 28], suggesting a broader social phenomenon. A possible reason for the differential trends observed between younger and older individuals lies in the differing mechanisms through which education could influence health at various stages of life. Over the life course, the effect of educational level on health is not necessarily linear, and depends on many age, period and cohort related factors [56, 57]. Education provides individuals with better access to healthcare, healthier lifestyles, and resources for managing chronic conditions [57] which positively and directly impact their health, especially as they age. In addition, education plays a role in enhancing cognitive resilience [58, 59], weakening the adverse effects of aging on functionality. On the other hand, for younger individuals, the potential exposure to social and economic stressors such as economic constraints, work-life imbalance, poor working conditions and inequalities could increase their vulnerability. Research suggests that in younger cohorts, mental health has been deteriorating over time [60, 61], which could be driven by the challenges of a competitive job market and academic pressures. Moreover, individuals working in lower socioeconomic positions have been reported to often experience greater physical and mental health challenges, partly due to adverse working conditions such as higher levels of effort-reward and demand-control imbalances [62]. All these issues could have an impact on overall wellbeing and affect lifestyle. As evidence suggests, adverse lifestyle related factors such as sedentary behavior [63] and obesity [38, 64] have been increasing in younger and middle aged adults. Moreover, the reported rise in childhood and adolescent overweight obesity over time, especially among lower socioeconomic groups [65], may have contributed to the adverse health trends among younger adults, as the increase in severe limitations could be a manifestation of cumulative effects of health behaviours established earlier in life. Moreover, younger adults may be particularly vulnerable to emerging health challenges associated with earlier onset of chronic conditions, such as type 2 diabetes [66]. These factors may help explain why activity limitations are increasing in this subgroup, despite the buffering effect of educational expansion, highlighting their vulnerability.

The study also revealed significant educational gradients in activity limitations, a finding consistent with numerous studies highlighting educational inequalities in health outcomes, including GALI [3, 6, 67]. Given the well-established role of education as a key resource for enhancing health, this result was expected. However, it is notable that educational inequalities in daily activity limitations have been widening over time, particularly among middle-aged individuals. Several studies have shown similar trends in widening educational inequalities in health-related outcomes, such as obesity, with socially disadvantaged individuals experiencing a larger share of the increase in rates compared to their higher socioeconomic status (SES) counterparts [64, 68, 69]. One explanation for this disparity is the possible worsening of lifestyle risk factors, such as poor nutrition and lack of physical activity. As these trends seem to be moving in a negative direction due to rising obesity trends [38, 64], individuals with lower levels of education may be more strongly affected, potentially due to limited resources or access to information that could help mitigate these risk factors. Since limitations in daily activities reflect the overall health status, which can be significantly influenced by other health indicators such as obesity, this explanation can also be applied to the widening inequalities observed in GALI. Moreover, the fact that middle-aged individuals, especially women, were particularly affected by the widening of educational inequalities in (severe) limitations could also be explained by the *digital divide* due to digitalization and technology, especially in occupational contexts. The digital divide refers to the gap between individuals or groups regarding the access to, literacy and use of technologies [70]. A European study suggests that even in countries which are leading in digital development, there are internal disparities in digital use with regard to educational attainment [71]. Against this background, it could be speculated that individuals with lower education are not benefitting equally from digital resources, growing the gap between educational groups with respect to health outcomes. Likely, the changing nature of work, with an increasing shift towards digitalization and automation, may disadvantage individuals without higher educational qualifications, who might find it more difficult to adapt to new technologies. This in turn could weaken the capability of individuals with lower SES compared to their higher SES counterparts to cope with lifestyle and health challenges.

### Strengths and limitations

This study is one of the rare studies examining effects of educational expansion on health and one of the first to consider self-perceived activity limitations against this background. The study is done using German nationally representative samples over a period of 17 years in total. Still, the results of the study should be viewed in light of several limitations associated with the survey design. Sampling bias associated with CATI, reporting bias, interviewer-respondent interaction as well as interviewing skills of interviewers are potential limitations that cannot be ruled out completely. In addition, a typical limitation is that severely ill individuals could be less likely to participate in surveys, leading to a potential underestimation of the prevalence of severe activity limitations. Moreover, the study considered school educational level as categorized according to the specific German schooling system. Additional investigations considering occupational education or international standard classifications of education such as ISCED could be beneficial to allow for international comparisons. Moreover, an additional limitation could be the possible shift in the perceptions or assessment of self-perceived activity limitations due to changing levels of health awareness over time. Similarly, age groups or gender differences in perceptions, expectations as well as assessment of activity limitations could also exist, partly explaining differences among genders and age groups. Thus, it would be beneficial to expand the analyses using additional health indicators. Moreover, the COVID-19 pandemic represents a contextual factor that may have introduced temporary disruptions when examining long term trends in education-related health inequalities. While our analysis distinguished pre- and post-pandemic survey waves, it did not explicitly model the pandemic as a structural break. Recent evidence from Germany shows that COVID-19 intensified income inequalities in mental health, where it was associated with a decline in mental health among regular, fluctuating and low household income trajectories, but not among high household income trajectories [72]. Moreover, pandemic-induced psychosocial stress has been shown to have significantly mediated educational differences in mental health–related quality of life [73]. Against this background, some fluctuations observed in our results may partly reflect pandemic-related dynamics.

## Conclusions

In conclusion, educational expansion has been demonstrated to have a positive association with individuals' health, highlighting the importance of efforts to increase educational levels. This emphasizes the value of educational reforms aimed at improving access to education, which can have broad health benefits. Further studies are needed to examine whether the observed mediating role of educational expansion reflects a causal process by investigating the underlying mechanisms. The study identified younger individuals as a particularly vulnerable group, suggesting that further investigation into the factors contributing to the deterioration of self-perceived activity limitations in this particular group is crucial for the development of effective health interventions. Additionally, middle-aged individuals are increasingly affected by rising educational inequalities, which further emphasizes the need to explore the underlying causes of this widening gap. In this context, it is essential to examine potential contributing factors such as social stressors and the digital divide, as these may play a significant role in exacerbating educational disparities and their subsequent impact on health outcomes.

## Supplementary Information

Additional file 1.

Additional file 2.

## Data Availability

Access restrictions exist on the data underlying the results. The dataset cannot be made publicly available because the informed consent from study participants does not cover public sharing. The minimal dataset underlying the results is archived at the Department of Medical Sociology of the Hannover Medical School and can be accessed by researchers upon justified request.

## Abbreviations

GALI: Global Activity Limitation Indicator
GEDA: Gesundheit in Deutschland Aktuell (German Health Update)
NDE: Natural Direct Effect
NIE: Natural Indirect Effect
RII: Relative Index of Inequality
RKI: Robert Koch Institute
SII: Slope Index of Inequality
TE: Total Effect
