# Supplementary material for: Is educational expansion beneficial for health? A counterfactual mediation analysis of its impact on trends of daily activity limitations among different age groups in Germany
Source: Arch Public Health. 2026 Aug 25;84:191. doi: 10.1186/s13690-026-02051-6 (PMC13505053; doi:10.1186/s13690-026-02051-6)
Supplement: Supplementary file 1 [file 13690_2026_2051_MOESM1_ESM.docx]

**Additional file 1**

**Manuscript: Is educational expansion beneficial for health? A counterfactual mediation analysis of its impact on trends of daily activity limitations among different age groups in Germany**

Authors: Batoul Safieddine, Johannes Beller, Stefanie Sperlich

Mediation model for the counterfactual analysis to examine mediation effects of educational expansion on trends of activity limitations
