# Supplementary material for: Is educational expansion beneficial for health? A counterfactual mediation analysis of its impact on trends of daily activity limitations among different age groups in Germany
Source: Arch Public Health. 2026 Aug 25;84:191. doi: 10.1186/s13690-026-02051-6 (PMC13505053; doi:10.1186/s13690-026-02051-6)
Supplement: Supplementary file 2 [file 13690_2026_2051_MOESM2_ESM.docx]

**Additional file 2**

**Manuscript: Is educational expansion beneficial for health? A counterfactual mediation analysis of its impact on trends of daily activity limitations among different age groups in Germany**

Authors: Batoul Safieddine, Johannes Beller, Stefanie Sperlich

**Additional table 1.** Odds ratios (OR) and confidence intervals (CI) displaying trends of the three levels of school education in the adult population in Germany, stratified by gender and three age groups. Examined using logistic regression analyses, adjusted for age within each age group and weighted according to population weights.

|  |  |  | **18-39 years** n (men)=19340  n (women)=20022 | | **40-64 years** n (men)=127461 n (women)=98668 | | **65+ years** n (men)=240860 n (women)=337892 | |
| --- | --- | --- | --- | --- | --- | --- | --- | --- |
|  |  |  | **OR** | **CI** | **OR** | **CI** | **OR** | **CI** |
| **Men** | **Low Education** | **2008/2009** | 1 | - | 1 | - | 1 | - |
|  |  | 2012/2013 | 0.80* | 0.67 - 0.97 | 0.77** | 0.67 - 0.88 | 1.03 | 0.86 - 1.22 |
|  |  | 2019/2020 | 0.67* | 0.52 - 0.86 | 0.74** | 0.62 - 0.87 | 0.60** | 0.50 - 0.73 |
|  |  | 2020/2021 | 0.73 | 0.50 - 1.07 | 0.57** | 0.44 - 0.75 | 0.62** | 0.48 - 0.80 |
|  |  | 2023/2024 | 0.80* | 0.65 - 0.98 | 0.61** | 0.53 - 0.71 | 0.52** | 0.44 - 0.62 |
|  | **Middle Education** | **2008/2009** | 1 | - | 1 | - | 1 | - |
|  |  | 2012/2013 | 1.01 | 0.88 - 1.17 | 1.04 | 0.93 - 1.17 | 0.85 | 0.69 - 1.04 |
|  |  | 2019/2020 | 1.06 | 0.88 - 1.29 | 1.06 | 0.92 - 1.23 | 1.49** | 1.22 - 1.82 |
|  |  | 2020/2021 | 1.04 | 0.77 - 1.39 | 1.32* | 1.06 - 1.64 | 1.33* | 1.02 - 1.75 |
|  |  | 2023/2024 | 1.08 | 0.92 - 1.25 | 1.26* | 1.10 - 1.43 | 1.58** | 1.31 - 1.90 |
|  | **High Education** | **2008/2009** | 1 | - | 1 | - | 1 | - |
|  |  | 2012/2013 | 1.24* | 1.09 - 1.41 | 1.27** | 1.15 - 1.41 | 1.22* | 1.04 - 1.43 |
|  |  | 2019/2020 | 1.40** | 1.18 - 1.67 | 1.43** | 1.26 - 1.62 | 1.44** | 1.23 - 1.69 |
|  |  | 2020/2021 | 1.35* | 1.04 - 1.75 | 1.42** | 1.17 - 1.72 | 1.56** | 1.28 - 1.96 |
|  |  | 2023/2024 | 1.22* | 1.06 - 1.40 | 1.41** | 1.26 - 1.58 | 1.62** | 1.40 - 1.87 |
| **Women** | **Low Education** | **2008/2009** | 1 | - | 1 | - | 1 | - |
|  |  | 2012/2013 | 0.76* | 0.62 - 0.93 | 0.77** | 0.67 - 0.88 | 0.92 | 0.79 - 1.07 |
|  |  | 2019/2020 | 0.51** | 0.37 - 0.70 | 0.48** | 0.40 - 0.57 | 0.45** | 0.38 - 0.52 |
|  |  | 2020/2021 | 0.45* | 0.27 - 0.75 | 0.52** | 0.39 - 0.70 | 0.43** | 0.34 - 0.54 |
|  |  | 2023/2024 | 0.54** | 0.42 - 0.69 | 0.41** | 0.35 - 0.49 | 0.44** | 0.38 - 0.51 |
|  | **Middle Education** | **2008/2009** | 1 | - | 1 | - | 1 | - |
|  |  | 2012/2013 | 0.81* | 0.71 - 0.92 | 0.99 | 0.89 - 1.10 | 0.97 | 0.82 - 1.14 |
|  |  | 2019/2020 | 0.80* | 0.66 - 0.98 | 1.08 | 0.95 - 1.22 | 1.83** | 1.56 - 2.15 |
|  |  | 2020/2021 | 0.79 | 0.58 - 1.08 | 0.96 | 0.79 - 1.17 | 1.89** | 1.50 - 2.37 |
|  |  | 2023/2024 | 0.71** | 0.61 - 0.84 | 1.10 | 0.99 - 1.23 | 1.84** | 1.59 - 2.13 |
|  | **High Education** | **2008/2009** | 1 | - | 1 | - | 1 | - |
|  |  | 2012/2013 | 1.46** | 1.29 - 1.65 | 1.38** | 1.25 - 1.52 | 1.39** | 1.18 - 1.63 |
|  |  | 2019/2020 | 1.92** | 1.60 - 2.31 | 2.05** | 1.83 - 2.30 | 2.13** | 1.82 - 2.49 |
|  |  | 2020/2021 | 1.99** | 1.50 - 2.65 | 2.06** | 1.70 - 2.49 | 2.00** | 1.62 - 2.47 |
|  |  | 2023/2024 | 2.10** | 1.81 - 2.43 | 2.21** | 1.99 - 2.45 | 2.08** | 1.81 - 2.40 |

***p<0.05; **p<0,005**

**Additional table 2.** Odds ratios (OR) and confidence intervals (CI) displaying trends of activity limitations in the adult population in Germany, stratified by gender and three age groups. Examined using logistic regression analyses, adjusted for age within each age group and weighted according to population weights.

|  |  | **18-39 years** n (men)=19340 n (women)=20022 | | **40-64 years** n (men)=127461 n (women)=98668 | | **65+ years** n (men)=240860 n (women)=337892 | |
| --- | --- | --- | --- | --- | --- | --- | --- |
|  |  | **OR** | **CI** | **OR** | **CI** | **OR** | **CI** |
| **Men** | **2008/2009** | 1 | - | 1 | - | 1 | - |
|  | 2012/2013 | 1.21 | 0.98 - 1.50 | 1.17* | 1.03 - 1.32 | 0.97 | 0.81 - 1.17 |
|  | 2019/2020 | 0.87 | 0.67 - 1.14 | 0.99 | 0.84 - 1.16 | 0.75* | 0.62 - 0.90 |
|  | 2020/2021 | 1.17 | 0.80 - 1.73 | 1.07 | 0.83 - 1.36 | 0.72* | 0.57 - 0.92 |
|  | 2023/2024 | 1.47* | 1.21 - 1.94 | 1.30* | 1.08 - 1.56 | 0.81* | 0.66 - 0.99 |
| **Women** | **2008/2009** | 1 | - | 1 | - | 1 | - |
|  | 2012/2013 | 1.02 | 0.85 - 1.23 | 1.08 | 0.97 - 1.22 | 0.97 | 0.82 - 1.13 |
|  | 2019/2020 | 1.18 | 0.92 - 1.53 | 1.03 | 0.90 - 1.18 | 0.68** | 0.58 - 0.80 |
|  | 2020/2021 | 0.95 | 0.64 - 1.41 | 1.03 | 0.83 - 1.29 | 0.70* | 0.56 - 0.88 |
|  | 2023/2024 | 1.66** | 1.29 - 2.15 | 1.28* | 1.09 - 1.49 | 0.85 | 0.72 - 1.02 |

***p<0.05; **p<0.005**

**Additional table 3.** Odds ratios (OR) and confidence intervals (CI) displaying trends of activity limitations in the adult population in Germany, stratified by educational level, gender and three age groups. Examined using logistic regression analyses, adjusted for age within each age group and weighted according to population weights.

|  |  |  | **18-39 years** n (men)=19340 n (women)=20022 | | **40-64 years** n (men)=127461 n (women)=98668 | | **65+ years** n (men)=240860 n (women)=337892 | |
| --- | --- | --- | --- | --- | --- | --- | --- | --- |
|  |  |  | **OR** | **CI** | **OR** | **CI** | **OR** | **CI** |
| **Men** | **Low Education** | **2008/2009** | 1 | - | 1 | - | 1 | - |
|  |  | 2012/2013 | 1.78* | 1.12 - 2.83 | 1.21 | 0.95 - 1.53 | 0.96 | 0.74 - 1.27 |
|  |  | 2019/2020 | 0.67 | 0.34 - 1.34 | 1.30 | 0.97 - 1.76 | 0.81 | 0.60 - 1.09 |
|  |  | 2020/2021 | 0.96 | 0.37 - 2.48 | 1.02 | 0.62 - 1.68 | 0.73 | 0.48 - 1.10 |
|  |  | 2023/2024 | 1.36 | 0.73 - 2.55 | 1.68* | 1.16 - 2.44 | 0.89 | 0.63 - 1.26 |
|  | **Middle Education** | **2008/2009** | 1 | - | 1 | - | 1 | - |
|  |  | 2012/2013 | 1.38 | 0.98 - 1.93 | 1.22 | 0.99 - 1.49 | 1.25 | 0.88 - 1.79 |
|  |  | 2019/2020 | 1.27 | 0.83 - 1.94 | 0.87 | 0.67 - 1.12 | 1.07 | 0.75 - 1.53 |
|  |  | 2020/2021 | 1.78 | 0.95 - 3.33 | 1.25 | 0.84 - 1.84 | 0.92 | 0.58 - 1.47 |
|  |  | 2023/2024 | 1.72* | 1.07 - 2.77 | 1.30 | 0.95 - 1.77 | 1.03 | 0.69 - 1.55 |
|  | **High Education** | **2008/2009** | 1 | - | 1 | - | 1 | - |
|  |  | 2012/2013 | 0.90 | 0.67 - 1.21 | 1.19 | 0.99 - 1.43 | 0.84 | 0.66 - 1.08 |
|  |  | 2019/2020 | 0.80 | 0.56 - 1.14 | 1.03 | 0.81 - 1.32 | 0.59** | 0.46 - 0.75 |
|  |  | 2020/2021 | 0.96 | 0.58 - 1.61 | 1.14 | 0.79 - 1.64 | 0.77 | 0.57 - 1.04 |
|  |  | 2023/2024 | 1.59* | 1.12 - 2.58 | 1.21 | 0.96 - 1.52 | 0.77* | 0.60 - 0.99 |
| **Women** | **Low Education** | **2008/2009** | 1 | - | 1 | - | 1 | - |
|  |  | 2012/2013 | 0.91 | 0.56 - 1.49 | 1.22 | 0.95 - 1.56 | 1.13 | 0.91 - 1.41 |
|  |  | 2019/2020 | 1.46 | 0.72 - 2.97 | 1.70* | 1.22 - 2.36 | 0.78 | 0.61 - 1.01 |
|  |  | 2020/2021 | 1.53 | 0.56 - 4.21 | 1.52 | 0.88 - 2.63 | 0.80 | 0.57 - 1.12 |
|  |  | 2023/2024 | 2.40* | 1.06 - 5.42 | 2.52** | 1.62 - 3.92 | 0.99 | 0.75 - 1.31 |
|  | **Middle Education** | **2008/2009** | 1 | - | 1 | - | 1 | - |
|  |  | 2012/2013 | 1.06 | 0.80 - 1.42 | 1.16 | 0.98 - 1.37 | 0.75* | 0.57 - 0.98 |
|  |  | 2019/2020 | 1.35 | 0.88 - 2.08 | 1.07 | 0.87 - 1.31 | 0.66* | 0.51 - 0.86 |
|  |  | 2020/2021 | 0.86 | 0.41 - 1.79 | 0.97 | 0.69 - 1.37 | 0.71 | 0.49 - 1.03 |
|  |  | 2023/2024 | 1.66* | 1.02 - 2.70 | 1.24 | 0.98 - 1.56 | 0.78 | 0.60 - 1.03 |
|  | **High Education** | **2008/2009** | 1 | - | 1 | - | 1 | - |
|  |  | 2012/2013 | 1.15 | 0.90 - 1.46 | 0.99 | 0.84 - 1.17 | 0.89 | 0.66 - 1.19 |
|  |  | 2019/2020 | 1.29 | 0.92 - 1.81 | 0.88 | 0.73 - 1.06 | 0.74* | 0.56 - 0.98 |
|  |  | 2020/2021 | 1.15 | 0.68 - 1.96 | 1.11 | 0.82 - 1.49 | 0.77 | 0.54 - 1.11 |
|  |  | 2023/2024 | 2.00** | 1.47 - 2.72 | 1.20 | 0.99 - 1.45 | 1.07 | 0.81 - 1.42 |

***p<0.05; **p<0.005**
